# The best-laid plans go oft awry: synaptogenic growth factor signaling in neuropsychiatric disease

**DOI:** 10.3389/fnsyn.2014.00004

**Published:** 2014-03-18

**Authors:** Aislinn J. Williams, Hisashi Umemori

**Affiliations:** ^1^Department of Psychiatry, University of MichiganAnn Arbor, MI, USA; ^2^Molecular and Behavioral Neuroscience Institute, University of MichiganAnn Arbor, MI, USA; ^3^Department of Neurology, F.M. Kirby Neurobiology Center, Harvard Medical School, Boston Children's HospitalBoston, MA, USA

**Keywords:** synapse, synaptogenesis, growth factor, psychiatry, mental illness

## Abstract

Growth factors play important roles in synapse formation. Mouse models of neuropsychiatric diseases suggest that defects in synaptogenic growth factors, their receptors, and signaling pathways can lead to disordered neural development and various behavioral phenotypes, including anxiety, memory problems, and social deficits. Genetic association studies in humans have found evidence for similar relationships between growth factor signaling pathways and neuropsychiatric phenotypes. Accumulating data suggest that dysfunction in neuronal circuitry, caused by defects in growth factor-mediated synapse formation, contributes to the susceptibility to multiple neuropsychiatric diseases, including epilepsy, autism, and disorders of thought and mood (e.g., schizophrenia and bipolar disorder, respectively). In this review, we will focus on how specific synaptogenic growth factors and their downstream signaling pathways might be involved in the development of neuropsychiatric diseases.

## Introduction

Neuropsychiatric diseases are increasingly recognized to have developmental origins. Some of these illnesses, such as autism and ADHD, must be diagnosed based on symptoms identified during early childhood (Association, 2013). Others, such as bipolar disorder and schizophrenia, are usually diagnosed in adulthood, but are recognized to have some manifestations in childhood as well (Martin and Smith, 2013; Schulz et al., 2014). Although these illnesses were initially studied in isolation from each other, there is increasing evidence that these clinically disparate diseases may have common genetic origins (Smoller and Finn, 2003; Lichtenstein et al., 2010; Sullivan et al., 2012; Cross-Disorder Group of the Psychiatric Genomics et al., 2013). To take this idea further, if these diseases begin early in development and have identifiable common genetic origins, it is possible, and perhaps even likely, that perturbations in some common developmental pathways may be involved in their pathogenesis.

**Figure 1:**
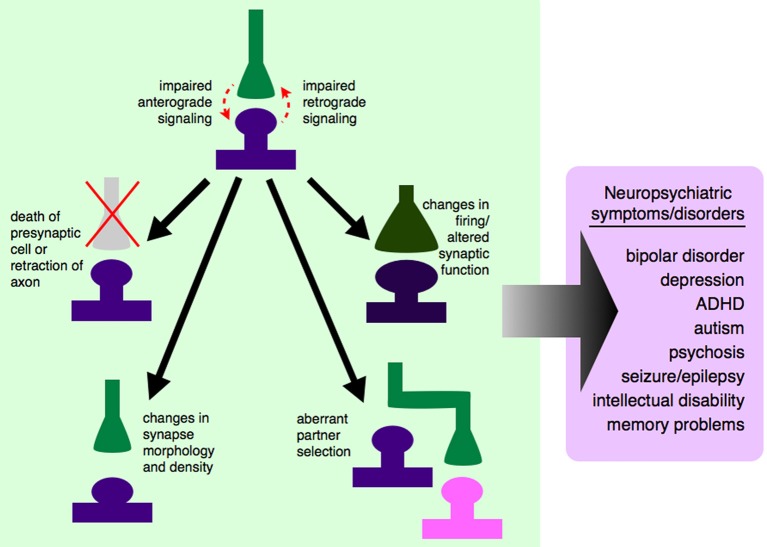
**Dysregulated growth factor signaling can lead to synaptic-level defects and neuropsychiatric disease**. Synaptogenic growth factors signal in both anterograde and retrograde directions, depending on the specific growth factor involved. If this signaling is disrupted, due to genetic mutations, changes in expression level, or changes in secretion pattern, synapses will not be established properly. Some growth factors exert trophic functions at the level of the synapse, and dysregulated signaling could lead to the death of the presynaptic cell or retraction of that axon from its appropriate postsynaptic partner. Even if the presynaptic axon is not retracted, impaired growth factor signaling between synaptic partners could cause changes in synaptic morphology and density, and ultimately to a non-functional synapse. Impaired signaling could also lead an axon to bypass its correct partner entirely and establish a synapse with a non-preferred partner, leading to aberrant neuronal circuitry. If appropriate signals are not passed between pre- and postsynaptic cells, this could lead to changes in action potential firing rates and altered communication between cells. It is still unclear which of these processes contribute to which neuropsychiatric diseases, although there are data to support neuron and synapse loss in certain cortical and hippocampal areas in mood disorders like depression and bipolar disorder (Manji et al., 2001; Stockmeier et al., 2004; Stockmeier and Rajkowska, 2004), aberrant brain connectivity in autism (Chung et al., 2013; Lynch et al., 2013; Uddin et al., 2013), and aberrant feed-forward loops (Yilmazer-Hanke et al., 2007) and neuronal circuitry (Aliashkevich et al., 2003) in epilepsy.

One major set of signaling molecules that are important in neural development are synaptogenic growth factors. These growth factors, including brain-derived neurotrophic factor (BDNF), the fibroblast growth factor (FGF) family, Wnts, and insulin-like growth factors (IGFs), are important not only in cell fate specification and neurogenesis, but specifically in the formation and maintenance of synapses (Vicario-Abejon et al., 1998; Barros et al., 2009; Terauchi et al., 2010; Guillemot and Zimmer, 2011; Corvin et al., 2012; Rosso and Inestrosa, 2013). Appropriate partnering of pre- and postsynaptic neurons is critical for the establishment of individual neuronal circuits, which in turn is the fundamental basis of overall wiring of the functional brain. Problems in these synaptogenic signaling pathways, which could occur either due to mutations in individual growth factors or their receptors, or inappropriate conduction of those signals through intracellular signaling pathways, could lead to abnormal connections between neurons or aberrant neuronal circuitry (Figure 1).

Several lines of evidence suggest that synaptogenic growth factors are involved in the pathogenesis of neuropsychiatric diseases. First, it is known that many mouse models with mutations in synaptogenic growth factors or their receptors have behavioral abnormalities, which may be analogous to neuropsychiatric disease in humans. For example, mice lacking FGF7 are predisposed to epilepsy in a kindling protocol (Terauchi et al., 2010). Second, some humans with mutations in growth factors have observable behavioral and cognitive problems. For example, people with a valine to methionine substitution at position 66 (V66M) in the proBDNF polypeptide have impaired episodic memory and increased risk of mood disorders (Egan et al., 2003; Schumacher et al., 2005). Finally, there is growing evidence that maintenance of proper networks and synaptogenesis and plasticity are impaired in neuropsychiatric illnesses (Brennand et al., 2011; Uddin et al., 2013), and growth factors are known to have a major role in all of these processes.

**Figure 2:**
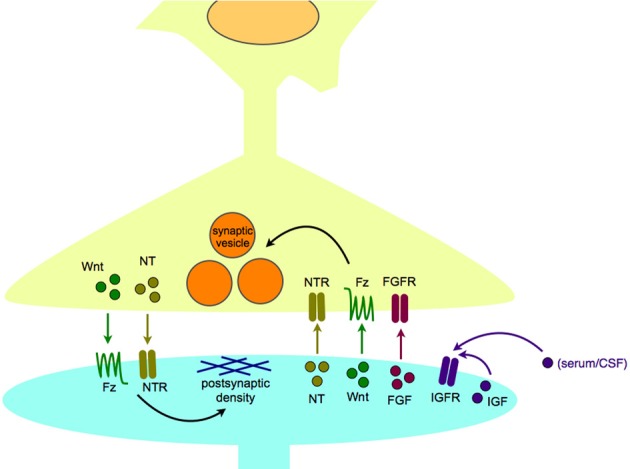
**Synaptogenic growth factors in normal synapse formation and maintenance**. Synaptogenic growth factors, including neurotrophins, Wnts, and FGFs, are secreted from the postsynaptic cell to induce appropriate differentiation of the presynaptic terminal, including clustering of synaptic vesicles. In turn, growth factors, including neurotrophins, and Wnts, can be released from the presynaptic cell to organize the differentiation of the postsynaptic density. IGFs are thought to act in a paracrine or autocrine fashion, and may be able to bind receptors within the synapse as well as at extrasynaptic sites. Abbreviations used: FGF, fibroblast growth factor; FGFR, fibroblast growth factor receptor; Fz, Frizzled receptor; IGF, insulin-like growth factor; IGFR, insulin-like growth factor receptor; NT, neurotrophin; NTR, neurotrophin receptor.

We propose that the critical stage of interest for studying these illnesses is during synaptogenesis, as this is when neurons are wired together to form functional circuits. For our purposes, “synaptogenesis” includes synapse development, maturation, and maintenance, as these steps are all essential for a mature, functional synapse. It is important to note that synapse maturation and modulation occur throughout life, and are likely to contribute to variations in disease presentation as development progresses. For example, FGF2 has been hypothesized as an “on-line” modulator of mood and anxiety in adults (Turner et al., 2006). Synaptogenic growth factors are released from both the pre- and postsynaptic neurons to assist synaptogenesis (Figure 2). Other developmental processes, such as neurogenesis and programmed cell death are also important in brain development, and occur throughout life; their potential contributions to the pathogenesis of neuropsychiatric diseases have been reviewed elsewhere (Margolis et al., 1994; Mennerick and Zorumski, 2000; Gigante et al., 2011; Petrik et al., 2012).

There are many molecules that act as synaptogenic growth factors in the brain. The most well-studied of these is BDNF, which has been linked to multiple neuropsychiatric diseases including bipolar disorder, depression, and schizophrenia (Neves-Pereira et al., 2002, 2005; Schumacher et al., 2005). The FGFs are a large family of growth factors, which are important in many processes throughout development. FGFs have recently been shown to be important in the development of glutamatergic and GABAergic synapses (Flajolet et al., 2008; Stevens et al., 2010; Terauchi et al., 2010) and have been implicated in a wide number of neuropsychiatric diseases (Evans et al., 2004; Perez et al., 2009; Terwisscha Van Scheltinga et al., 2010; Yamanaka et al., 2011; Turner et al., 2012). Wnts and their receptors have been implicated in learning and memory (Tabatadze et al., 2012; Fortress et al., 2013), autism (Wassink et al., 2001), and some forms of epilepsy (Lako et al., 1998). Although the insulin-like growth factor (IGF) family of factors and receptors was previously recognized primarily for its importance in neurogenesis, development, and aging, this family is also now known to have roles in cortical plasticity (Tropea et al., 2006) and memory (Chen et al., 2011). Other families of growth factors have also been shown to be active in synaptogenesis and brain development, such as the TGFβ, GDNF, and EGF/neuregulin families (Mei and Xiong, 2008; Paratcha and Ledda, 2008; Williamson and Hiesinger, 2008; Van Kesteren et al., 2008; Krieglstein et al., 2011). The contributions of growth factors are summarized in Table 1.

**Table 1:**
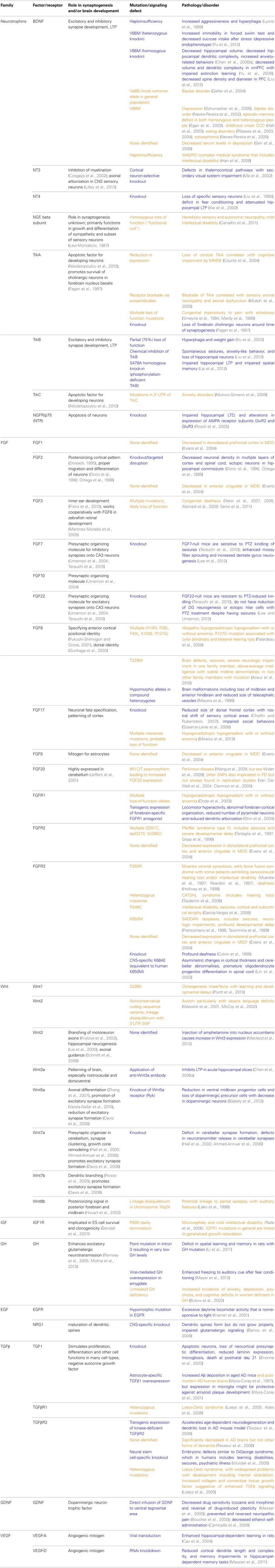
**Growth factors and their receptors in synaptogenesis and neuropsychiatric disease**.

*Growth factors proposed to be involved in brain development are listed, along with available evidence for their involvement in neuropsychiatric disease. Evidence from animal models is listed in blue, evidence from studies in humans or tissue derived from humans is listed in orange. Abbreviations used: 3 '-UTR, 3 '-untranslated region; AD, Alzheimer disease; AMPA, α-amino-3-hydroxy-5-methyl-4-isoxazolepropionic acid; BDNF, brain-derived neurotrophic factor; CA3, cornu ammonis area 3; CATSHL, camptodactyly, tall stature, scoliosis, and hearing loss; CNS, central nervous system; DG, dentate gyrus; EGF, epidermal growth factor; ES cell, embryonic stem cell; Fz, Frizzled receptor; GDNF, glial-derived neurotrophic factor; GH, growth hormone; GluR, AMPA glutamate receptor subunit; JNK, c-Jun N-terminal kinase; LTP, long-term potentiation; MDD, major depressive disorder; miR, microRNA; MMSE, mini-mental status exam; NGF, nerve growth factor; NRG, neuregulin; NT-3, neurotrophin-3; NT-4, neurotrophin-4; OCD, obsessive compulsive disorder; PD, Parkinson disease; PFC, prefrontal cortex; PTZ, pentylenetetrazol; RNAi, RNA interference; Ryk, atypical receptor tyrosine kinase; SADDAN, severe achondroplasia with developmental delay and acanthosis nigricans; SNP, single nucleotide polymorphism; TGF, transforming growth factor; vmPFC, ventromedial prefrontal cortex; WAGRO, wilms tumor, aniridia, genitourinary anomalies, mental retardation, and obesity syndrome*.

Growth factor signaling between pre- and postsynaptic neurons ensures that proper connections between both individual neurons and brain regions are made. Here we describe how dysregulation of these systems may lead to neuropsychiatric disease. Since many of these synaptogenic growth factors promote intracellular signaling through common signal transduction pathways, it is possible that modulation of one or a few of these pathways could lead to significant improvement of clinical symptoms.

## BDNF and the neurotrophin family of growth factors

The neurotrophin family of growth factors includes BDNF, pro-BDNF, NGF, NT-3, and NT-4. Neurotrophins bind to the Trk family of receptors, as well as the p75 receptor, to activate multiple intracellular signaling cascades. BDNF binds primarily to TrkB, NGF to TrkA, NT-3 to TrkC and TrkB, and NT-4 primarily to TrkB. All neurotrophins bind with relatively low affinity to the p75 receptor, and proBDNF binds only the p75 receptor. When neurotrophins bind to Trks, they support the survival and growth of neurons. Neurotrophins secreted from the postsynaptic cell promote the survival and health of the presynaptic neuron, and maintain a synapse between the two cells. One exception to this rule is the binding of proBDNF to p75, which is usually a pro-apoptotic signal to neurons (Teng et al., 2005). BDNF may also participate in postsynaptic organization (Johnson-Venkatesh and Umemori, 2010; Yoshii et al., 2011), but it is unclear whether this is independent of its presynaptic organizational activities.

BDNF signaling plays a major role in CNS synaptogenesis. It is involved in development of both excitatory and inhibitory synapses (Itami et al., 2000; Fiorentino et al., 2009), and is important for strengthening excitatory synapses through long-term potentiation, a form of cellular and network learning and memory (Minichiello, 2009). BDNF is released from neurons both constitutively and in an activity-dependent fashion (Farhadi et al., 2000; Zha et al., 2001; Egan et al., 2003), and its activity-dependent expression is important for the development and maintenance of cortical inhibitory synapses (Hong et al., 2008). Mice with a hypomorphic or null TrkB allele in hindbrain neurons demonstrate impaired climbing fiber pruning at the climbing fiber-Purkinje cell synapse (Johnson et al., 2007), suggesting that TrkB signaling is important for developmental synaptic pruning, possibly in an activity-dependent fashion. BDNF also plays a role in shaping dendritic morphology, which is an important aspect of synaptogenesis. Mice homozygous (knock-in) for the V66M BDNF mutation show decreased cortical spine density and diameter (Liu et al., 2012), as well as decreased hippocampal and cortical dendritic complexity (Chen et al., 2006b; Yu et al., 2009). BDNF is upregulated in mouse hippocampus under conditions of environmental enrichment (Hu et al., 2013), which is known to enhance dendritic arborization (Turner et al., 2003) and number of hippocampal synapses (Gogolla et al., 2009; Babic and Zinsmaier, 2011). BDNF may have differential effects depending on where its mRNA is translated in the cell; recent evidence shows that somatic BDNF is important for dendritic spine formation, whereas dendritic BDNF expression is important for spine head growth and spine pruning (Orefice et al., 2013). Other neurotrophins, including NT-3 and NT-4, have not been shown conclusively to be involved in synaptogenesis; these do have importance in neurogenesis and other aspects of CNS development (Table 1).

BDNF has been implicated in the pathogenesis of multiple neuropsychiatric diseases, including depression (Schumacher et al., 2005), schizophrenia (Zintzaras, 2007), and Rett syndrome, a severe developmental disorder with autistic features (Larimore et al., 2009; Zeev et al., 2009). The V66M mutation in BDNF impairs activity-dependent release of BDNF in hippocampal cultures and is associated with impaired episodic memory, both in patients with schizophrenia and people without neurologic or psychiatric illness (Egan et al., 2003). Interestingly, this mutation may be both a risk factor for depression (Schumacher et al., 2005) and a protective factor against bipolar disorder (Geller et al., 2004), although not all genetic studies in humans support these associations (Neves-Pereira et al., 2002). Data from post-mortem patient tissue supports changes in mRNA and protein levels of BDNF and TrkB in patients with mood and psychotic disorders (Issa et al., 2010; Thompson Ray et al., 2011; Tripp et al., 2012; Qi et al., 2013). Data from animal models with deficits in neurotrophin signaling support the links between BDNF signaling and behavior. Mice lacking TrkB in forebrain neurons show impaired spatial learning, delay in fear conditioning, and impaired hippocampal LTP (Minichiello et al., 1999), as well as behavioral rigidity when faced with changing environmental conditions (Vyssotski et al., 2002). Another group, using the same forebrain-specific TrkB knockout mice, observed increased behavioral hyperactivity and impulsivity (Zorner et al., 2003). Mice with reduced BDNF expression levels display increased alcohol consumption (Hensler et al., 2003; McGough et al., 2004). The same is observed in mice when trkB expression levels are reduced (Jeanblanc et al., 2006). Although it is unknown whether NT-4 functions specifically in synaptogenesis, NT-4 null mice have deficits in fear conditioning and hippocampal LTP (Xie et al., 2000), which may have implications for human anxiety and cognitive disorders.

## Fibroblast growth factors and their receptors

The FGF family includes 22 FGF genes, which are clustered into groups based on phylogenetic similarity and receptor specificity (Umemori, 2009). Among them, there are 4 FGF homologous factors (originally called FGFs 11–14, now known as FHFs), which are solely intracellular signaling molecules and do not bind to FGF receptors (FGFRs). Other FGFs bind to FGFRs, of which there are 4 genes that can be alternatively spliced into multiple receptor subtypes (Umemori, 2009). FGF signaling is important in organogenesis and growth throughout development (Beenken and Mohammadi, 2009). In the CNS, FGFs have many functions, including neurogenesis, fate specification, and neuronal survival (Dono, 2003; Mason, 2007). FGFs also play roles in axon guidance and target recognition. For example, FGF8 has been shown to be an axon guidance molecule for trochlear nerve axons in a cultured rat midbrain explant model of neuronal pathfinding (Irving et al., 2002), and FGF2 gradients help retinal ganglion cell axons find their targets in the optic tectum in developing Xenopus (McFarlane et al., 1995). Although some FGFs act by an endocrine mechanism in the periphery, (such as FGF19, FGF21, and FGF23), FGFs that are active in the CNS are released by postsynaptic cells to stimulate presynaptic organization (Terauchi et al., 2010), and therefore act primarily by local mechanisms within the CNS.

Evidence is accumulating for the importance of FGFs in synaptogenesis. In cultured rat hippocampal neurons, addition of FGF2 to the culture medium generates an increase in excitatory synapses via a MAPK-dependent mechanism (Li et al., 2002). In cultured neurons, FGF7 and FGF22 function as presynaptic organizers (Umemori et al., 2004; Terauchi et al., 2010). FGF7-null mice have a deficit in hippocampal inhibitory synapse formation while FGF22-null mice are deficient in hippocampal excitatory synapses (Terauchi et al., 2010), consistent with the roles of FGF7 and FGF22 in presynaptic organization (Umemori et al., 2004). Other FGFs, including FGFs 4, 6, and 9 also promote synaptic vesicle clustering in cultured neurons (Umemori et al., 2004), but their roles in synaptogenesis *in vivo* are unknown.

The evidence linking FGFs and behavioral abnormalities is growing. FGF7-null are prone to develop epilepsy after kindling, while FGF22-null are resistant to seizure induction (Terauchi et al., 2010), providing a link between synaptogenic defects and a neurobehavioral phenotype. Mice overexpressing FGF21 primarily in the liver, which is known to function in metabolism and insulin sensitivity, also show dysregulation in circadian rhythms, which is a common feature of mood disorders (Bookout et al., 2013); it is unknown whether these mice have other behavioral abnormalities consistent with mood alterations. It is also unknown whether FGF21 plays a role in synaptogenesis, although it is known to cross the blood-brain barrier (Bookout et al., 2013). Mice globally lacking FGF17 have impaired social interactions, a key diagnostic feature of autism (Scearce-Levie et al., 2008). Interestingly, FGF17 was found to induce neurite branching in cultured neurons (Umemori et al., 2004), suggestive that abnormal connectivity between neurons may underlie these behavioral changes in FGF17-null mice. Peripheral administration of FGF2 to rats with endogenously high levels of anxiety was found to reduce anxiety-like behaviors (Perez et al., 2009), while lentiviral shRNA-mediated knockdown of FGF2 in rat hippocampus increased anxiety-like behaviors (Eren-Kocak et al., 2011). Although no studies of FGF expression in anxiety disorder patients have been published, the body of literature supports the idea that FGF2, if not other FGFs, is an important regulator of many emotional states. Alterations in FGF expression in humans have also been associated with depression (Evans et al., 2004), substance abuse (Turner et al., 2012), and schizophrenia (Terwisscha Van Scheltinga et al., 2010). Mutations in FGFR2 are causative for Pfeiffer Syndrome, some severe forms of which manifest intellectual disability (Priolo et al., 2000; Shotelersuk et al., 2002). There are multiple other examples of FGFs and FGFRs linked to neuropsychiatric disease, which are detailed in Table 1. Overall, the data underscore the importance of normal FGF signaling both for normal synapse formation and normal neuropsychiatric functioning.

## Wnt signaling molecules and their receptors

Wnts are a family of 19 highly-conserved secreted signaling glycoproteins that play important roles in embryogenesis and fate specification in early development. When they bind to their receptors, the Frizzled proteins and LRP coreceptors, they can trigger several different types of intracellular signaling pathways. The best characterized intracellular signaling pathway is the Wnt/Frizzled/β-catenin/GSK3-β pathway, also known as the canonical pathway. Wnts 1, 2, 3a, 7a, and 7b generally signal through the canonical pathway. There are also several non-canonical pathways that have been identified which do not signal via β-catenin, the most well-studied of which are the planar cell polarity (PCP) and the Wnt/calcium pathways. Wnts 4a and 5a signal through the PCP pathway, which is involved in neuronal migration as well as cell polarity (Okerlund and Cheyette, 2011). The Wnt/calcium pathway is important for control of calcium release from the endoplasmic reticulum (ER) for calcium-dependent intracellular signals (De, 2011). There are also a number of other Wnt signaling pathways, but these are generally less well-understood (Niehrs, 2012). Although Frizzled and LRP are the most well-studied receptors for Wnts, Wnts are also known to bind to many other cell surface receptors, including Ryk, ROR2, and others (Niehrs, 2012).

Wnt signaling pathways have many roles in CNS synaptogenesis, and can both increase or decrease synapse formation depending on the Wnt pathways and cell types involved. The role of Wnts in non-mammalian and peripheral nervous system synaptogenesis has been reviewed extensively elsewhere (Park and Shen, 2012; Poon et al., 2013). Wnt7a is a retrograde signal derived from cerebellar granule cells to presynaptic mossy fiber terminals in the cerebellum (Hall et al., 2000). Wnt7a binds to Dvl1, a mouse homolog of Disheveled, and induces clustering of synapsin I and axon growth cone remodeling (Hall et al., 2000; Ahmad-Annuar et al., 2006). In mice globally lacking either Wnt7a or Dvl1, there are deficits in cerebellar synapse formation, while mice null for both Wnt7a and Dvl1 have an additional defect in neurotransmitter release at mossy fiber-granule cell synapses (Hall et al., 2000; Ahmad-Annuar et al., 2006). Wnt7a also has a role in synaptic differentiation in the hippocampus, particularly enhancing the number and strength of excitatory synapses (Davis et al., 2008; Ciani et al., 2011); this is also true for Wnt7b (Davis et al., 2008). Wnt5a has been shown to increase the formation of glutamatergic synapses and maturation of dendritic spines in cultured neurons via a calcium-dependent mechanism (Varela-Nallar et al., 2010). However, in a separate study, application of Wnt5a to neuronal cultures resulted in a decrease in glutamatergic synapses (Davis et al., 2008), suggesting that Wnt5a effects may be dependent on culture conditions or downstream signaling pathways (canonical vs. non-canonical). Taken together, the data demonstrate the importance of Wnt signaling in synaptogenesis in both pre- and postsynaptic compartments.

Although many knockout mouse models for Wnts have been developed, most do not survive embryogenesis (Uusitalo et al., 1999; Van Amerongen and Berns, 2006), and therefore cannot be assessed for behavioral phenotypes. However, there are mouse models where other mediators of Wnt signaling have been genetically manipulated, which implicate Wnt signaling in behavior. Mice null for Dvl1 have diminished social interactions, a core feature of autism, as well as abnormal prepulse inhibition, which is observed in both autism and schizophrenia (Lijam et al., 1997). These mice also have deficits in hippocampal dendritic branching and cerebellar synaptogenesis (Lijam et al., 1997; Rosso et al., 2005). Forebrain-specific reduction of expression of β-catenin, the putative downstream signaling molecule for Dvl1, generates subtle behavioral changes in the tail suspension test, a depression-like endophenotype (Gould et al., 2008). The lack of similar behavioral deficits between the forebrain-specific β-catenin knockout and Dvl1-null mice could be due to the fact that the β-catenin knockout was limited to the forebrain, whereas the synaptic changes noted in Dvl1-null mice are primarily noted in hippocampus and cerebellum, or may be attributable to the multiplicity of downstream effectors of Wnt signaling. Another way to modulate Wnt signaling is by overexpressing Axin, a scaffolding protein that negatively regulates Wnt signaling. When mice overexpressing Axin are trained in a fear-conditioning paradigm, they exhibit an increase in freezing to contextual conditioning as well as changes in cued fear conditioning, suggestive that alterations in Wnt signaling could increase anxiety-related behaviors (Kim et al., 2011).

Wnts have been implicated in multiple genetic studies of human neuropsychiatric disease. Some data suggest that mutations in Wnt2 are linked with forms of autism with severe language deficits (Wassink et al., 2001), although not all studies have confirmed this association (McCoy et al., 2002). Interestingly, the *CHD8* gene has been identified in multiple genetic studies of autism and related neurodevelopmental disorders (Neale et al., 2012; O'Roak et al., 2012a,b; Talkowski et al., 2012). The CHD8 protein binds β-catenin and negatively regulates Wnt/β-catenin signaling (Nishiyama et al., 2012). Wnts also can activate the retinoid-related orphan receptor alpha, RORA, which has been implicated by GWAS in several neuropsychiatric diseases, including autism (Nguyen et al., 2010; Sarachana and Hu, 2013), bipolar disorder (Le-Niculescu et al., 2009; but see McGrath et al., 2009), depression (Terracciano et al., 2010; Utge et al., 2010), and PTSD (Logue et al., 2013). Additionally, both lithium and valproic acid, medications commonly used to treat neuropsychiatric diseases, are known to inhibit GSK3β, a downstream effector of the canonical Wnt signaling pathway (Lucas and Salinas, 1997; Hall et al., 2002), and lithium treatment in mice activates Wnt signaling in various regions of the brain including amygdala and hippocampus (O'Brien et al., 2004).

## Insulin-like growth factors and their receptors

IGFs are peptide growth factors identified based on their similarity to the peptide hormone, insulin. The family consists of two growth factor ligands (IGF1 and IGF2), two receptors (IGF1R and IGF2R), and multiple IGF binding proteins (IGFBPs) and IGFBP-related proteins (Fernandez and Torres-Aleman, 2012). IGF1 is a neurotrophic factor that enhances the survival of neurons in culture (Meyer-Franke et al., 1995; Arnaldez and Helman, 2012; O'Kusky and Ye, 2012). IGF2 has also been implicated in neurogenesis, synaptogenesis, myelination, and dendritic branching (Agis-Balboa et al., 2011; Fernandez and Torres-Aleman, 2012; Schmeisser et al., 2012). The mechanism of IGF1 action on neurons may be both endocrine and autocrine, as it circulates in the bloodstream and can cross the blood-brain barrier, in addition to being secreted locally by neurons (Nunez et al., 2003) (Figure 2). IGF2 may also serve a neurotrophic function, at least for young hippocampal neurons, since increasing IGF2/IGFBP7 signaling via a fear-conditioning paradigm in mice leads to enhanced survival of newborn hippocampal neurons (Agis-Balboa et al., 2011).

The primary physiologic receptor for the IGFs is IGF1R, although IGF1 can also bind the insulin receptor. Like many growth factor receptors, IGF1R is a receptor tyrosine kinase, and when bound by IGF1, can activate several different intracellular cascades (Arnaldez and Helman, 2012). IGF2R can bind IGF2, but not IGF1. IGF2R is thought primarily to sequester IGF2 at the cell surface, and in most cases this binding does not generate transmembrane signals. IGFBPs regulate IGF activity by binding to IGFs and IGF1R, and this binding can inhibit or facilitate the binding of IGFs to IGF1R, or prolong the half-life of IGFs, depending upon the IGF/IGFBP pair and the specific microenvironment (O'Kusky and Ye, 2012).

IGF1 is widely expressed throughout the brain throughout development (Garcia-Segura et al., 1991), and IGF1 is upregulated in neurons during the developmental periods associated with dendritic maturation and synapse formation (Bondy, 1991). Application of IGF1 to cultured cortical neurons causes an increase in puncta containing PSD-95 and synapsin, but not puncta containing gephyrin, suggestive that IGF1 treatment increases the number of excitatory rather than inhibitory synapses in the cortex (Corvin et al., 2012). Interestingly, in mice modeling a severe form of autism, Rett syndrome, treatment with an active IGF1 peptide fragment partially restores spine density, synaptic function, PSD-95 localization and levels, and synaptic plasticity (Tropea et al., 2009). IGF2 is expressed in neurons and may localize to synaptic sites, and application of IGF2 to cultured hippocampal neurons causes an increase in spine formation via an IGF2R-dependent mechanism (Schmeisser et al., 2012), in contrast to previous data suggesting that IGF2R functions only as a reservoir to bind IGF2 at the cell surface. IGF1R is found in both pre- and postsynaptic areas in certain hypothalamic nuclei and the cerebellum (Garcia-Segura et al., 1997), suggestive that IGF signaling may play roles in both pre- and postsynaptic organization. IGF2R also localizes to postsynaptic densities (Schmeisser et al., 2012).

Animal models have demonstrated the importance of IGFs in normal synaptogenesis as well as neuropsychiatric disease. In rat pups, environmental enrichment during youth is also known to reduce anxiety-like behaviors during adulthood, but this effect of environmental enrichment is lost when IGF1 activity is blocked by systemic injection of blocking peptide during environmental enrichment. Interestingly, IGF1 injection during youth mimics the anxiolytic effects of environmental enrichment when the rats reach adulthood (Baldini et al., 2013). Blockade of IGF1 during youth, and the concomitant increase in anxiety-like behaviors in adulthood, is correlated with increased hippocampal IGF1R expression at postnatal day 12 in rats and increased glucocorticoid receptor expression at postnatal day 60 (Baldini et al., 2013). Interestingly, IGF1 infusion into the CSF of adult rats improved their performance on both cognitive and affective reactivity tasks (Markowska et al., 1998).

There are limited data from humans on the potential role of IGF signaling in neuropsychiatric disease, but there are some lines of evidence that implicate IGF signaling may be important. Lithium is one of the most effective treatments available for bipolar disorder, and it is known to inhibit GSK3β (Hedgepeth et al., 1997; Chalecka-Franaszek and Chuang, 1999). In patient-derived lymphoblastoid cell lines, bipolar disorder patients who respond to lithium have higher levels of IGF1 than bipolar disorder patients who do not respond to lithium (Squassina et al., 2013). This suggests that IGF1 may act upstream of GSK3β in modulating lithium response (Cui et al., 1998; Chalecka-Franaszek and Chuang, 1999). There is also significant evidence that insulin and IGF signaling promote the aging process in many animals (Bartke, 2008; Kenyon, 2010), raising the intriguing possibility that age-related cognitive decline may be mediated by the effects of insulin and IGFs on transcription factors and synapse function.

## Synaptogenic growth factor signaling pathways

There is significant crossover in the intracellular downstream signaling pathways activated by synaptogenic growth factors. These pathways include (a) the MAPK/ERK pathway, (b) the PI3K/Akt pathway, and (c) the PLC/IP3/CAMK pathway. Significantly, all of these pathways have been implicated in several different neuropsychiatric diseases. We will address the evidence linking each individual pathway to synaptogenesis and disease, and then present a model that may help explain how these systems are linked in disease pathogenesis.

### The MAPK/ERK pathway

The MAPK/ERK pathway is a common signal transduction pathway for many synaptogenic growth factors, including BDNF, FGFs, some Wnts, and IGF1 (Easton et al., 1999; Perron and Bixby, 1999; Quevedo et al., 2000; Bikkavilli et al., 2008). This signaling cascade begins when a synaptogenic growth factor binds its receptor, often itself a receptor tyrosine kinase except in the case of some Wnt receptors, and activates it. This results in binding of intracellular signaling proteins, which ultimately activate MAPK, which activates ERK. ERK can activate multiple transcription factors, including CREB, RSK, and myc. There are many ways in which alterations in this signaling pathway can contribute to neuropsychiatric disease. A mutation in RSK2, one of the downstream effector molecules of this pathway, can cause Coffin-Lowry syndrome, an X-linked form of severe intellectual disability (Morice et al., 2013). Mutations of the RSK2 gene in humans are associated with smaller volumes of hippocampus, cerebellum and temporal lobe, while a mouse model of Coffin-Lowry syndrome lacking Rsk2 demonstrates defects in hippocampal spine morphology and hippocampus-dependent learning (Morice et al., 2013). There is also evidence that environmental stressors can alter the MAPK/ERK pathway. In rhesus monkeys who were abused or neglected by their mothers during childhood, decreased CSF serotonin metabolites were correlated with both activated p38 MAPK in serum monocytes as well as increased risk of anxiety behaviors, delayed social development and reduced exploration as adolescents (McCormack et al., 2006; Sanchez et al., 2007).

### The PI3K/Akt pathway

Another critical intracellular signaling pathway, the PI3K/Akt pathway, is activated when synaptogenic growth factor receptors phosphorylate PI3K. PI3K activation then leads to phosphorylation of Akt. Akt can translocate into the nucleus to regulate other transcription factors, leading to changes in levels of synaptic proteins, and can also activate mTOR (mammalian target of rapamycin) and thereby indirectly influence the growth and survival of cells. In neurons, the PI3K/Akt pathway is activated by growth factors including BDNF and IGF1 (Stroppolo et al., 2001; Bondy and Cheng, 2004; Li and Thiele, 2007). One study suggests that exogenous FGF1 may activate this pathway in astrocytes (Ito et al., 2013), although whether this is also the case for neurons is unknown. There is some evidence that this pathway can be activated by FGFR2 in oligodendrocytes independent of any FGF ligand at all (Bryant et al., 2009). This pathway is also activated by FGF7 in lung tissue (Ray et al., 2003), but it is not known whether FGF7 can also activate this pathway in neurons. Environmental enrichment, which is known to have multiple beneficial effects on anxiety-like behaviors in rodents, upregulates the Akt pathway and leads to downregulation of GSK3β (Hu et al., 2013). Akt itself has been implicated as a risk factor for schizophrenia susceptibility, as a specific *AKT1* haplotype causes decreased Akt levels and is associated with illness (Emamian et al., 2004). Another Akt family member, AKT3, has been shown to be important in some cases of brain malformation and epilepsy (Poduri et al., 2012b), linking this pathway to multiple aspects of brain development, including circuit formation, circuit activity, and neuronal survival. Finally, the specific serotonin reuptake inhibitor, fluoxetine, increases phosphorylation of Akt as well as ERK in rat neural stem cells (Kitagishi et al., 2012; Huang et al., 2013). The fact that fluoxetine and other medications in its class are useful for a wide variety of neuropsychiatric illnesses outside of depression lends further support to the idea that these illnesses may have common origins.

### The PLC/IP3/CAMK pathway

A third common signal transduction pathway activated by many synaptogenic growth factors and implicated in neuropsychiatric disease is the PLC/IP3/CAMK pathway. It is activated by BDNF, many FGFs, and some Wnts (Klint and Claesson-Welsh, 1999; Reichardt, 2006). It may be induced by IGF1, although it is unknown whether this is via direct IGFR1 activation of PLC or if this occurs indirectly (Chattopadhyay and Carpenter, 2002). In this pathway, activation of receptor tyrosine kinases by extracellular binding of synaptogenic growth factors leads to activation of phospholipase C (PLC, most commonly PLCγ1) and generation of the second messenger IP3. IP3 diffuses to the ER where it binds to its receptor, IP3R. IP3R is a calcium channel that releases calcium from the ER. When released into the cytosol, calcium can bind to a number of calcium-dependent proteins, such as calmodulin, which activates a number of important intracellular enzymes, including the calmodulin-dependent kinases (CAMKs). CAMKs are important effector molecules for a number of neuronal functions, including long-term potentiation (Sanhueza et al., 2007) and calcium-response element (CRE)-dependent transcription (Kang et al., 2001). One particular CAMK protein, Camk2B, is expressed widely in the CNS, and levels of *CAMK2B* mRNA were found to be upregulated 2-fold in the frontal cortex of post-mortem schizophrenia patient brains compared to control brains (Novak et al., 2000). In a single patient, a point mutation in *CAMK2G* (R29P) was associated with a number of phenotypic abnormalities, including severe intellectual disability (De Ligt et al., 2012). Mice lacking Camk4 have deficits in fear learning, with corresponding reductions in phosphorylated CREB in brain areas associated with fear memory after training (Wei et al., 2002). One of the upstream signaling factors in this pathway, PLCβ1, has been implicated in severe forms of epilepsy (Kurian et al., 2010; Poduri et al., 2012a). All of these lines of evidence point to the importance of this pathway in normal neural development and function, and there is clear evidence of synaptic dysfunction and behavioral phenotypes when these pathways are altered.

## Crosstalk between signaling pathways

**Figure 3:**
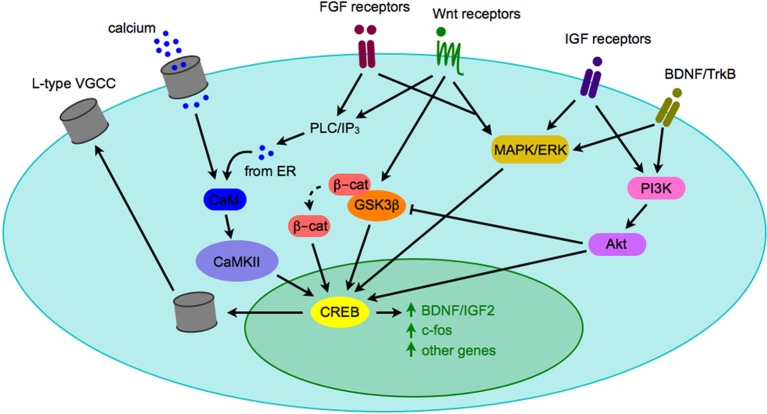
**Crosstalk between signaling pathways implicated in pathogenesis of neuropsychiatric diseases**. When synaptogenic growth factors bind to their respective receptors, they can trigger a number of intracellular signaling cascades. Depicted here are the PLC/IP3/CAMK pathway, the MAPK/ERK pathway, and the PI3K/Akt pathway. Although there are a number of downstream effectors of each pathway, they may converge on the CREB transcription factor and gene expression, including expression of genes that modify neuronal activity, such as BDNF and the L-type VGCC. Although only one cell is depicted here, the model could apply to a presynaptic or postsynaptic cell. Abbreviations used: β-cat, β-catenin; BDNF, brain-derived neurotrophic factor; CaM, calmodulin; CaMKII, calmodulin-dependent kinase II; CREB, calcium response element binding protein; ER, endoplasmic reticulum; ERK, extracellular signal-regulated kinase; FGF, fibroblast growth factor; GSK3β, glycogen synthase kinase 3β; IGF, insulin-like growth factor; IP3, inositol 1,4,5-trisphosphate; MAPK, mitogen-activated protein kinase; PI3K, phosphatidylinositide 3-kinase; PLC, phospholipase C; TrkB, tyrosine receptor kinase B; VGCC, voltage-gated calcium channel.

Most synaptogenic growth factors can activate multiple downstream signaling pathways depending on which receptor they bind, and in which cell type the receptor is expressed. Due to the fact that activated growth factor receptors can bind promiscuously to various intracellular second messengers, it is unlikely that any single growth factor signaling cascade will account for all of the phenotypes observed in a given neuropsychiatric disease. It is far more likely that the complex interplay of a number of signaling pathways will generate an observable phenotype, such as autism or depression. However, all three pathways described above have intermediary signaling molecules (ERK, Akt, and CaMKII) that can activate cAMP/calcium-response element binding protein (CREB) and lead to CRE-dependent transcription of genes (Figure 3). CREB-mediated transcription is critical for expression of a number of genes, including some synaptogenic growth factors as well as c-fos and other activity-dependent genes (Benito and Barco, 2010). CREB may serve as a key integrator of signals of neuronal activity, such as NMDA receptor activation-mediated calcium influx, with synaptogenic growth factor signaling (such as the cascades described above). Activation of CREB then leads to transcription of activity-dependent genes that play roles in synaptogenesis. One such activity-dependent gene is the L-type voltage-gated calcium channel (VGCC), which has recently been the focus of much interest as SNPs within the alpha subunit of one L-type VGCC has been implicated as a risk factor in multiple neuropsychiatric diseases (Andreassen et al., 2013; Cross-Disorder Group of the Psychiatric Genomics et al., 2013).

Another possible cellular focus for synaptogenic growth factor signal integration is the WAVE regulatory complex (WRC), a large five-subunit complex that controls actin cytoskeleton dynamics (Pollitt and Insall, 2009). Recently, two papers were published which describe how cell surface receptors containing a WRC interacting receptor sequence (WIRS) domain interact with the WRC and the actin cytoskeleton to direct synapse formation and changes in neuronal morphology including axonal branching (Chen et al., 2014; Chia et al., 2014). Many synaptic proteins have potential WIRS domains, including some synaptogenic growth factor receptors (Chen et al., 2014). The WAVE complex might be another major intergrator of synaptogenic growth factor signaling in neurons.

Crosstalk between multiple growth factor pathways occurs as well, further underlining how interconnected these systems are in the brain. For example, Wnt signaling triggers transcription of FGF4 in tooth development (Kratochwil et al., 2002), sequential signaling by Wnt3a and FGF8 are required to induce dorsalization during brain development (Gunhaga et al., 2003), and both FGF19 and Wnt8C signaling are required for successful inner ear development (Ladher et al., 2000). Cooperative signaling of the Wnt and FGF systems is also critical in spinal cord specification (Nordstrom et al., 2006). Recently, crosstalk between FGF and Wnt signaling in *C. elegans* sensory organs was described on a transcriptional level, where FGF activates the MAPK/ERK pathway and regulates a downstream Wnt effector molecule (Squarzoni et al., 2011). Therefore, it will be important to consider that modulation of a single synaptogenic growth factor or intracellular signaling pathway will likely affect other systems as well.

## Conclusions and future directions

Growth factor signaling between pre- and postsynaptic neurons is critical for proper connections between individual neurons, and for the development of appropriate brain circuitry. Synaptogenic growth factors play a key role in ensuring that synapses develop properly and are modulated appropriately over time, so that suitable emotional and behavioral responses to the environment are generated when necessary. As described, dysregulation of these systems may lead to inappropriate emotional and behavioral responses to either internal or external stimuli, which is associated with functional decline. Modulation of synapses over time is also critical for learning and memory when the environment changes, and dysfunction in these processes likely contributes to cognitive impairment. Ongoing synaptogenic dysregulation caused by defects in growth factor signaling may cause these illnesses not to improve (as in autism) or worsen and become increasingly difficult to treat (such as schizophrenia) over a patient's lifetime. Complicating this picture is the possibility that the specific functions of growth factors may change throughout development. Conversely, at different times throughout the life cycle, different growth factors may be required for similar functions. For example, at the neuromuscular junction (NMJ), laminin-β2 is a critical presynaptic organizer in the neonate, whereas collagen IV performs this function in the adult (Nishimune et al., 2004; Fox et al., 2007). Therefore, it is possible that the mutations or abnormalities in growth factors may only be relevant at specific developmental times, or in different locations, for specific neuropsychiatric diseases. Additionally, many other genetic risk factors for neuropsychiatric diseases are associated with synapse-specific proteins, including the synaptic scaffolding Shank proteins (Guilmatre et al., 2014), the synaptic adhesion molecules contactin/caspr and neurexin/neuroligin (Sudhof, 2008; Vernes et al., 2008; Kenny et al., 2013; Zuko et al., 2013), and proteins in the mTOR pathway, which is critical for synapse-specific protein synthesis (Hoeffer and Klann, 2010; Russo et al., 2012; Wong, 2013). These molecules and pathways may interact with the growth factor pathways (Patzke and Ernsberger, 2000; Iki et al., 2005; Hoeffer and Klann, 2010; Williams and Casanova, 2011; Russo et al., 2012; Wong, 2013; Bennett and Lagopoulos, 2014). The myriad ways in which these pathways may be linked requires further exploration.

Nevertheless, since many receptors for synaptogenic growth factors act through common intracellular signal transduction pathways, it may be that modulation of one or a few of these pathways could lead to significant resolution of clinical symptoms. In addition, growth factor binding proteins often act as regulators of growth factor binding and localization, which have the added benefit of functioning in the extracellular space rather than intracellular compartments. This could significantly reduce the difficulty of getting treatments to their target sites. Additionally, in the case of FGFs, heparan sulfate proteoglycans (HSPGs) are required for binding of FGFs to their receptors at high affinity (Klint and Claesson-Welsh, 1999). Modulation of certain HSPGs could alter FGF binding to particular FGFRs. Such an approach may also be possible with other synaptogenic growth factors.

### Conflict of interest statement

The authors declare that the research was conducted in the absence of any commercial or financial relationships that could be construed as a potential conflict of interest.
